# Vanillin derivatives as antiamnesic agents in scopolamine-induced memory impairment in mice

**DOI:** 10.1016/j.heliyon.2024.e26657

**Published:** 2024-02-19

**Authors:** Qamar Gul, Nasiara Karim, Mohammad Shoaib, Muhammad Zahoor, Mehboob Ur Rahman, Hayat Bilal, Riaz Ullah, Amal Alotaibi

**Affiliations:** a Department of Pharmacy, University of Malakand, Chakdara Dir Lower, KPK, Pakistan; b Department of Pharmacy, University of Peshawar, Peshawar, KPK, Pakistan; c Department of Biochemistry, University of Malakand Chakdara Dir Lower, KPK, Pakistan; d Department of Biotechnology, Abdul Wali Khan University Mardan, Mardan, KPK, Pakistan; e Medicinal Aromatic and Poisonous Plants Research Center College of Pharmacy, King Saud University, Riyadh, Saudi Arabia; f Department of Basic Science, College of Medicine, Princess Nourah Bint Abdulrahman University, 11671, Riyadh, Saudi Arabia

**Keywords:** Vanillin, Schiff bases, Acetylcholinesterase, Butyrylcholinesterase, Antioxidant, DPPH, Antiamnesic

## Abstract

Amnesia is a major health problem prevalent in almost every part of the world specifically in old age peoples. Vanillin analogues have played an important role in the field medicines. Some of them have been documented to be promising inhibitors of cholinesterases and could therefore, be used as antidepressant, anti-Alzheimer and as neuroprotective drugs. In this connection, the present study was designed to synthesize new vanillin analogues (**SB-1** to **SB-6**) of varied biological potentials. The synthesized compounds were investigated as inhibitors against acetylcholinesterase (AChE) and butyrylcholinesterase (BChE) enzymes and as scavengers of DPPH and ABTS free radicals followed by behavioural antiamnesic evaluation in mice. The compounds; **SB-1**, **SB-3**, **SB-4** and **SB-6** more potently inhibited AChE with IC_50_ values of 0.078, 0.157, 0.108, and 0.014 μM respectively. The BChE was more potently inhibited by **SB-3** with IC_50_ of 0.057 μM. Moreover, all of the tested compounds exhibited strong antioxidant potentials with promising results of **SB-3** against DPPH with IC_50_ of 0.305 μM, while **SB-5** was most active against ABTS with IC_50_ of 0.190 μM. The *in-vivo* studies revealed the improvement in memory deficit caused by scopolamine. Y-Maze and new object recognition test showed a considerable decline in cognitive dysfunctions. In Y-Maze test the spontaneous alteration of 69.44 ± 1% and 84.88 ± 1.35% for **SB-1** and 68.92 ± 1% and 80.89 ± 1% for **SB-3** at both test doses were recorded while during the novel object recognition test the Discrimination Index percentage of **SB-1** was more pronounced as compared to standard drug. All compounds were found to be potent inhibitors of AChE, BChE, DPPH, and ABTS *in vitro* however, **SB-1** and **SB-3** were comparatively more potent. **SB-1** was also more active in reclamation of memory deficit caused by scopolamine. **SB-1** and **SB-3** may be considered as excellent drug candidates for treating amnesia subjected to toxicological evaluations in other animal models.

## Highlights

• The synthesis of vanillin analogues with good yield and purity were performed.
• The synthesized compounds exhibited promising IC_50_ values against the tested cholinesterases.
• The synthesized analogues exhibited potential antioxidant activities against ABTS and DPPH with promising IC_50_ values.
• The vanillin derivatives were potent antiamnesic agents as predicted from *in-vivo* Y-maze and NORT models.

## 1 Introduction

Aromatic aldehydes-based Schiff bases are more stable as compared to aliphatic aldehydes and therefore, are versatile compounds of much economic importance. Schiff bases are a vast group of compounds characterized by the presence of a double bond linking carbon and nitrogen atoms, the versatility of which is generated in the many ways to combine a variety of alkyl or aryl substituents. Compounds of this type like Morphine, Demerol, Novocaine, Ephedra etc. are both found in nature and synthesized in the laboratory. By virtue of amine (-C=N) functionality, like other compounds, Schiff bases have exhibited broad range of biological effects such as antibacterial, antifungal, anticancer, herbicidal, and antileishmanial properties [[1], [2], [3]]. Apart from the mentioned biological potentials, the Schiff base complexes of Co (II), Fe (II), and Ru (II) (formed from hydroxyl benzaldehyde) have played a key role in the oxidation of cyclohexane into cyclohexanol and cyclohexanone, the compounds of much economic importance [4,5]. The antibiotic actions due to the azomethine (H–C=N-) group of Schiff bases have also been documented [6]. In a number of studies, antibacterial, anti-Alzheimer, anticancer, antileishmanial, anti-tuberculous, anticonvulsant, anti-inflammatory, antioxidant, antiviral, urease inhibitory, and pesticidal activities of Schiff bases and their metal complexes have been documented [[7], [8], [9], [10], [11]].

Alzheimer's disease (AD) is a complex condition in which irreversible neuronal death causes memory loss (amnesia) and cognitive impairment with other neuropsychiatric symptoms such as depression, apathy, anxiety, agitation, and hallucinations. AD is still a serious challenge for scientists around the globe that not only affecting the quality of an individual life but also have increased the burden on medical community, families and society [12]. AD being a learning and cognitive dysfunctions with behavioural turbulence and steady memory loss more drastically effect the individual life style, most effecting peoples with age 65 or more [13,14]. More than 50 million peoples are suffering from AD globally. Accumulation of β-amyloid plaques, highly phosphorylated tau proteins, neuronal damage due to free radicals and decrease in the concentration of essential neurotransmitters such as acetyl choline (ACh) and butyrylcholine (BCh) are the physiological changes associated with AD [[15], [16], [17]]. According to a reported study, the extracellular β-amyloid plaques (Aβ) and neurofibrillary tangles (NFTs) deposition due to hyperphosphorylation of tau protein plays a significant role in the development of AD [18]. Alzheimer's disease currently has no known cure, however there are therapies that can help with the symptom [19,20]. Inhibitors of acetylcholinesterase (AChE) and butyrylcholinesterase (BChE) are normally the drugs of choice to cure dementia and related diseases.

Plants based therapies are usually preferred due to low incidences of side effects. Vanillin, an important chemical component of vanilla beans, is commonly utilised as a food flavouring in food commodities. Vanillin is also a potential antioxidant that can potently scavenge the synthetic free radicals like ABTS and DPPH. Vanillin has been shown to inhibit mutagen induced-DNA damage, spontaneous mutation in bacteria and human cells by eliciting DNA repair [21], as well as having anti-cancer effects through mechanisms such as increased apoptosis and cell cycle arrest in melanoma, colon, and cervical cancer cells [22,23]. Shi et al. found that vanillin alleviated scopolamine-induced impairment by preventing the loss of immune-reactivity. Research studies have demonstrated that vanillin is effective in prevention and treatment of cognitive impairment and is a good candidate to be used clinically [24]. Beula et al. has reported Isatin (SWA) derivatives with anti-amnesic action [25,26]. In a number of studies vanillin Schiff bases have been found to have potent antioxidant and anticholinesterase activities (both in *in-vitro* and *in-vivo* studies).

A lot of research has been carried out on vanillin or its derivatives. Based on the reported potential of vanillin derivatives the present study was aimed to synthesize vanillin-based Schiff bases and evaluate them as inhibitors of AChE and BChE. The synthesized derivatives were also tested for their antioxidant potential. The behavioural studies were performed utilizing mice as animal model to decide whether these compounds could be used as potential drug candidates for the treatment of AD or not.

## 2 Materials and methods

### 2.1 Chemicals and instruments

The chemicals used in this study were of analytical grade and used as such without any further purification. They were purchased from Sigma Aldrich (St. Louis, Mo., USA), S.D Fine Chemicals (India) and Merck (Darmstadt, Germany). Commercially available TLC plates were used to monitor the progress of the reactions. Instrumental techniques like FTIR and NMR were used to characterize the synthesized compounds.

### 2.2 Synthesis of schiff bases

**Scheme 1:**
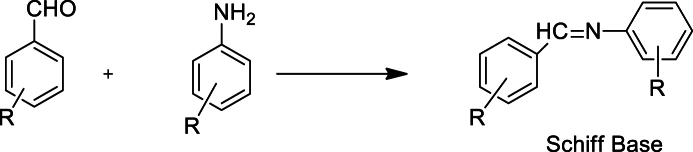
Synthesis of novel Schiff bases.

The synthesis of Schiff bases was carried out as given in Scheme 1. Aromatic aldehyde and aromatic amines were treated to get different substituted Schiff bases following the reported procedure in literature [27,28].

The synthesized derivatives were washed and recrystallized to get pure compounds which were then characterized by spectroscopic techniques like FTIR, ^1^H, and ^13^C NMR. The synthesized compounds were then stored in refrigerator till the commencement of pharmacological evaluations, *in vitro* and *in vivo*.

#### 2.2.1 Solid phase synthesis of schiff base compounds (method A)

Solid phase synthesis is considered to be a greener approach of synthesizing a number of valuable compounds of biological importance. Therefore, this approach has been followed in this study. The synthesized compounds are arbitrary presented as **SB1– SB6**.

Equimolar quantities of substituted benzaldehydes like 3-methoxy-4-hydroxybenzaldehyde, 3-hydroxy,4-methoxybenzaldehyde and 2-trifluoromethyl aniline, and 2-chloro-4-methyl aniline were mixed thoroughly and added with acetic acid (0.5 mL) which were then converted into paste using a mortar and pestle. After triturating for 50 min, the reaction progress was monitored using TLC plates. After confirmation of the reaction completion, the paste was added into 15 mL distilled water. The solution was filtered and subjected to recrystallization in ethanol to get the targeted compounds in pure state [29].

#### 2.2.2 Solution-phase synthesis (method B)

**Fig. 1:**
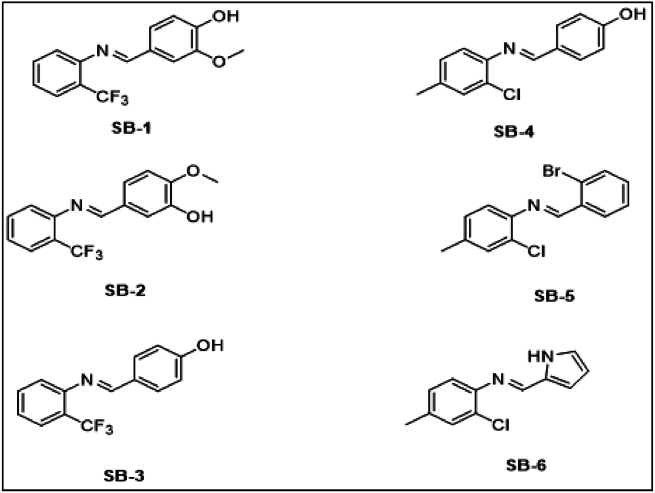
Structural representation of the synthesized novel Schiff bases.

In a 100 mL two necked round bottom flask equipped with a reflux condenser, on oil bath with magnetic stirrer and hot plate, added equimolar quantities of 3-methoxy-4-hydroxybenzaldehyde, 3-hydroxy, 4-methoxybenzaldehyde and 2-trifluoromethyl aniline, 2-chloro-4-methyl aniline in ethanol as solvent, with catalytic amount of acetic acid. The contents were mixed and stirred with occasional stirring for 4–6 h. The progress of reaction was checked through TLC. After completion of reaction, the solvent was evaporated under reduced pressure and the crude product was poured on to crushed ice. The solid obtained was filtered, washed with cold water, dried and finally recrystallized from n-hexane [30]. The chemical structures of the synthesized compounds are given in Fig. 1.

#### 2.2.3 Synthesis of (E)-2-methoxy-4-(((2-(trifluoromethyl)phenyl) imino) methyl)phenol (SB-1)

The compound (E)-2-methoxy-4-(((2-(trifluoromethyl) phenyl) imino) methyl) phenol (**SB-1**) was synthesized from 3-methoxy-4-hydroxybenzaldehyde and 2-trifluoromethyl aniline by both solid and solution phase methods as described above. The compound was further recrystallized from ethyl alcohol to afford pure solid product.

Yield: 75% (method A), 59% (method B). White crystalline solid M.P = 134–138 °C, Rf = 0.78 (Methanol: Chloroform, 1:19).

FTIR (cm^−1^): 3350 (OH), 2900–2980 (C–H, Ar), (1623 (C=N), 1588 (C=C, Ar).

^1^H NMR (500 MHz, CDCl_3_): *δ* (ppm): 8.28 (s, 1H, N=CH), 7.70 (s, 1H, Ar–H), 7.03 (d, 1H, *J* = *6.*2 Hz*,* Ar–H), 6.01 (d, 1H, *J* = *6.*4 Hz*,* Ar–H), 7.69–7.57 (m, 4H, Ar–H), 5.35 (bs, 1H, OH), 4.02 (s, 3H, OCH_3_) [89].

^13^C NMR (175 MHz, CDCl_3_): *δ* (ppm): 161.1 (N=CH), 151.2 (Ar-C-OH), 149.6, (Ar-C-O-CH3), 147.3(Ar-C-N), 133.4, 129.5, 126.3, 122.3 (Ar–C), 133.4, 127.5, 127.3, 121.3 (Ar–C), 124.9 (CF_3_), 56.2 (OCH_3_) [90].

#### 2.2.4 (E)-2-methoxy-5-(((2-(trifluoromethyl)phenyl)imino)methyl)phenol (SB-2)

The compound (E)-2-methoxy-5-(((2-(trifluoromethyl)phenyl)imino)methyl)phenol was synthesized from 3-hydroxy-4-methoxybenzaldehyde and 2-trifluoromethyl aniline per described detail of both solid and solution phase methods. The compound was further recrystallized from ethyl alcohol to afford pure solid product.

Yield: 67% (method 3.3.1), 51% (method 3.3.2). White crystalline solid, M.P = 122–126 °C. Rf = 0.73 (Methanol: Chloroform, 1:19).

FTIR (cm^−1^): 3376 (OH), 1656(C=N), 1583(C=C, Ar), 1511(C–O).

^1^H NMR (500 MHz, CDCl_3_): *δ* (ppm): 8.62 (s, 1H, N=CH), 7.53 (s, 1H, Ar–H), 7.31 (d, 1H, *J* = *6.*4 Hz*,* Ar–H), 6.92 (d, 1H, *J* = *6.*8 Hz*,* Ar–H), 7.61–7.01 (m, 4H, Ar–H), 5.32 (bs, 1H, OH), 3.81 (s, 3H, OCH_3_).

^13^C NMR (175 MHz, CDCl_3_): *δ* (ppm): 160.1 (N=CH), 151.1 (Ar-C-OH), 149.2 (Ar-C-O-CH3), 147.1 (Ar-C-N), 130.1, 129.3, 117.2, 112.3 (Ar–C), 133.1, 127.2, 127.1, 121.2 (Ar–C), 124.2 (CF_3_), 52.2 (OCH_3_).

#### 2.2.5 (E)-4-(((2-(trifluoromethyl) phenyl) imino)methyl)phenol (SB-3)

Following the general procedures as described above, the titled compound (E)-4-(((2-(trifluoromethyl) phenyl) imino) methyl)phenol (SB-3) was obtained in good yield.

Yield: 73% (method 3.3.1), 42% (method 3.3.2). Grey colour solid. M.P = 151–157 °C. Rf = 0.72 (Methanol: Chloroform, 1:19).

FTIR (cm^−1^): 3160 (OH), 1668 (C=N), 1580 (C=C, 1448 (C–F).

^1^H NMR (500 MHz, CDCl_3_): *δ* (ppm): 9.82 (s, 1H, N=CH), 7.53 (d, 2H, *J* = *8.*4 Hz*,* Ar–H), 6.85 (d, 2H, *J* = *6.*8 Hz*,* Ar–H), 7.61–7.01 (m, 4H, Ar–H), 5.32 (bs, 1H, OH).

^13^C NMR (175 MHz, CDCl_3_): *δ* (ppm): 160.1 (N=CH), 151.1 (Ar-C-OH), 147.1 (Ar-C-N), 130.1, 129.3, 117.2, 112.3 (Ar–C), 133.1, 127.2, 127.1, 121.2 (Ar–C), 124.2 (CF_3_).

#### 2.2.6 (E)-4-(((2-chloro-4-methylphenyl)imino)methyl)phenol (SB-4)

The compound (E)-4-(((2-chloro-4-methylphenyl)imino)methyl)phenol (**SB-4**)**,** was prepared from 4-hydroxybenzaldehyde and 2-chloro-4-methyl aniline by above described methods.

Yield: 70% (method 3.3.1), 48% (method 3.3.2). Brown colour solid, M.P = 161–164 °C. Rf = 0.72 (methanol: chloroform, 1:19).

FTIR (cm^−1^): 3081 (C–H, Ar), 1683 (C=N), 1511 (C–Cl, Ar), 1450 (C–Cl).

^1^H NMR (400 MHz, DMSO): *δ* (ppm): 10.16 (bs, 1H, OH), 8.37 (s, 1H, N=CH), 7.76 (t, 2H, *J* = *8.*4 Hz*,* Ar–H), 7.30 (s, 1H, Ar–H), 7.14 (d, 1H, *J* = *8.*0 Hz*,* Ar–H), 7.085 (d, 1H, *J* = 8 Hz, Ar–H), 6.89 (m, 2H, Ar–H), 2. 28 (s, 3H, CH3).

^13^C NMR (175 MHz, CDCl_3_): *δ* (ppm): 160.1 (N=CH), 156.1 (Ar-C-OH), 149.2 (Ar-C-N), 147.1 (Ar-C-N), 138.1, 130.3, 127.2, 123.3 (Ar–C), 133.1, 127.2, 127.1, 121.2 (Ar–C), 127.2 (Ar-C-Cl), 19.3 (CH_3_).

#### 2.2.7 (E)-N-(2-bromobenzylidene)-2-chloro-4-methylaniline (SB-5)

The compound (E)-N-(2-bromobenzylidene)-2-chloro-4-methylaniline (**SB-5**) was prepared from 2-bromobenzaldehyde and 2-chloro-4-methyl aniline as described.

Yield: 78% (method 3.3.1), 60% (method 3.3.2). Yellowish colour solid, M.P = 89–96 °C. Rf = 0.76 (methanol: chloroform, 1:19).

FTIR (cm^−1^): 3200 (C–H, Ar), 1610 (C=N), 1594 (C=C, Ar), 1412 (C–Cl).

^1^H NMR (500 MHz, CDCl_3_): *δ* (ppm): 8.62 (s, 1H, N=CH), 7.43 (s, 1H, Ar–H), 7.15 (d, 1H, *J* = 8 Hz, Ar–H), 7.11 (d, 1H, *J* = *6.*8 Hz Ar–H), 7.71–7.41 (m, 4H, Ar–H), 2.34 (s, 3 H, CH3). ^13^C NMR (175 MHz, CDCl_3_): *δ* (ppm): 159.8 (N=CH), 146.1 (Ar-C-N), 136.1, 132.3, 127.2, 121.3 (Ar–C), 138.1, 130.2, 127.1, 123.2 (Ar–C), 20.7 (CH_3_).

#### 2.2.8 (E)-N-((1H-pyrrol-2-yl) methylene)-2-chloro-4-methylaniline (SB-6)

The compound (E)-N-((1H-pyrrol-2-yl) methylene)-2-chloro-4-methylaniline (**SB-6**) was prepared from pyrrol-2-carboxyldehyde and 2-chloro-4-methyl aniline per used procedures.

Yield: 73% (method 3.3.1), 52% (method 3.3.2). Black colour solid, M. P = 105–112 °C. Rf = 0.73 (methanol: chloroform, 1:19).

FTIR (cm^−1^): 3100 (C–H, Ar), 1654 (C=N), 1572 (C=C, Ar), 1452 (C–Cl).

^1^H NMR (500 MHz, CDCl_3_): *δ* (ppm): 8.40 (s, 1H, N=CH), 6.95–6.15 (m, 3H, pyro-H), 7.31 (s, 1H, Ar–H), 7.15–7.11 (d, 2H, *J* = *6.*8 Hz*,* Ar–H), 5.02 (bs, 1H, NH), 2.31 (s, 3 H, CH3).

^13^C NMR (175 MHz, CDCl_3_): *δ* (ppm): 157.1 (N=CH), 141.1 (Ar-C-N), 127.2 (Ar-C-Cl), 147.1 (Ar-C-N), 130.1, 129.3, 117.2, 112.3 (Ar–C), 124.1, 119.2, 110.1, (pyrr-C), 19.9 (CH_3_).

### 2.3 Pharmacological evaluation

#### 2.3.1 Cholinesterases inhibition

To evaluate the therapeutic effectiveness of the compounds as inhibitors of cholinesterases, the reported procedures were followed (Elman's protocol) [31]. Enzymes acetylcholinesterase (EC 3.1.1.7) and butyrylcholinesterase (EC 3.1.1.8) were purchased from local market.

The acetylcholinesterase inhibition assay is based on the principle of the hydrolytic breakdown of substrate; acetylthiocholine iodide by acetylcholinesterase enzyme. The AChE acts on acetylthiocholine to thiocholine which then react with DTNB that's leads towards the formations of anion (5-thio-2-nitrobenzoate) a yellow colour product which is then assessed via UV visible spectrophotometer at 405 nm [32].

The AChE, Ellman's Reagent (DTNB) and acetylthiocholine iodide (ATChI, substrate), and the test compound solutions were made in different concentrations ranges from (0.01–1.0 μM) were incubated per detail given in the Ellman's protocol. Galantamine was employed as the reference drug [33,34]. IC_50_ of each Schiff bases were calculated using Microsoft Excel 2013 software.

In similar fashion following BChE, Ellman's Reagent (DTNB) and butyryl thiocholine iodide (BTChI, substrate), and the test compound Schiff bases solutions were made in different concentrations ranges from (0.01–1.0 μM) were incubated following the standard protocol. The absorbance at 405 nm was measured and the data were analysed in triplicate. Galantamine was employed as standard drug [33,34].

#### 2.3.2 Antioxidant activities

A slightly modified DPPH assay as described by Brand William was followed [35]. Initially, the absorbance of 3 mL from stock DPPH (20 mg/100 mL of methanol) was adjusted to 0.75 at 517 nm. The DPPH stock solution was covered and kept in the dark overnight to generate free radicals. About 2 mL of each dilution of different ranges (0.1–3.5 μM) was prepared from **SB1– SB6** stock (5 mg/5 mL of methanol) were mixed with 2 mL of pre-incubated DPPH stock solution and incubated for 15 min at room temperature. Ascorbic acid was used as a standard. The absorbance of the reaction mixtures was recorded at 517 nm and the % inhibition was calculated by Equation (1) [36]:

[1] $$\%\;\text{inhibition}=\frac{A-B}{A}\times 100$$

where A = absorbance of pure DPPH in oxidized form, B = absorbance of the sample, which

Was measured after 15 min of reaction with DPPH.

The antioxidant potential of the synthesized compounds were also determined using ABTS free radical following a reported standard protocol [37]. ABTS (7 mM) and K2S2O8 (2.45 mM) were mixed (in methanol) and put in the dark for 24 h for free radicals’ formation, which was then used as stock solution. The absorbance of 3 mL from the stock ABTS was adjusted to 0.75 at 745 nm, which was considered a control. About 300 μl of each of the serial dilutions (0.1–3.5 μM) of **SB1– SB6** and 3 mL of stock ABTS were mixed and incubated for 15 min at 25 °C, and their absorbance was measured at 745 nm. Ascorbic acid was used as a control. The scavenging activity was calculated by Equation (1).

### 2.4 *In vivo* behavioural studies

#### 2.4.1 Acute toxicity testing

Acute toxicity evaluation of the prepared compounds was performed using mice as experimental model following protocol as described by Lorke [37]. The protocol is implemented in two phases. In the first phase, the animals divided into three groups and each group containing three animals were given 100, 500 and 1000 mg/kg body weight doses of test compounds. The animals were kept under close observation for 24–72 h and their behaviour was monitored. The mortality if occurred was also noted. In the second phase animals were divided into groups of three and were administrated doses in higher concentrations (1250, 2500 and 5000 mg/kg) of test samples, then observed for 24–72 h followed by two weeks observation for behaviour as well as mortality. With the toxicity data at hand, effective doses (mg/kg b.w) were selected for behavioural studies.

#### 2.4.2 Animals and ethical approval

Albino mice (20–23 g b. w.) were brought from National Institute of Health, Islamabad, Pakistan, and the mice were retained in animal house at University of Malakand at Department of Pharmacy for acclimation. Normal pellet diet and water were provided to all the animals and were kept for 12 h of light-dark cycle under normal laboratory conditions (temperature in a range of 25 ± 2 °C, humidity of 56–66%). All protocols employed were approved from the Ethical Committee vide notification Pharm/EC-QRE/10–31/25 as per approved “Animal Bye-Laws 2008, Scientific Procedures Issue-I of the Malakand University” and the UK animal scientific procedure act (1986).

#### 2.4.3 *In-vivo* evaluation of anti-amnesic activity

After acclimatization, the animals were grouped into different groups consisting of 6 animals each. The group I received only intraperitoneal (i.p.) dose of scopolamine dissolved in normal saline at a dose of 2 mg/kg b.w before 30 min of the commencement of experiment. The scopolamine administration enhances cholinesterase activities which lead to cholinergic deficit ultimately resulting in amnesia. It also can induce amyloid β deposition, oxidative stress, synaptic dysfunction, and learning/memory impairment in experimental animals [38]. Group II received scopolamine along with galantamine as standard. The remaining groups received the **SB1-SB6** suspended in saline and Tween-80 at various doses (mg/kg b.w) along with scopolamine. The treatment was carried out continuously for 7 days and after treatment schedule, the animals were subjected to behavioural study using Y-maze and NORT.

##### 2.4.3.1 Y-maze spontaneous alternations

**Fig. 2:**
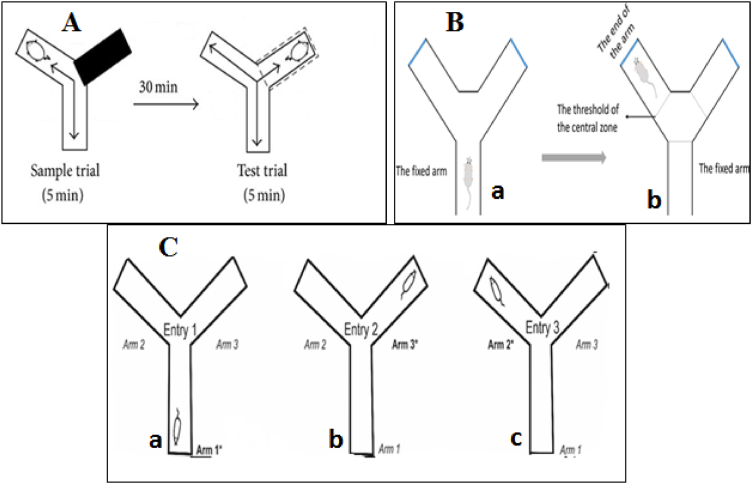
(A). Schematic drawings of the Y-maze and the experimental procedures. The sample trial and test trials were conducted for 5 min at a 30 min interval. **(B)** The Y-Maze test diagrammatic figure. (**a**) The mouse was positioned in the middle of the fixed arm and faced the maze's centre during the test. (**b**) A mouse was considered to have entered an arm when all four of its paws passed the threshold inside the arm and the mouse's snout was pointed in the direction of the end of the arm. (**C).** Alternations consist of single, uninterrupted entries into each arm of the y-maze. There are three steps involved in each alternation. The steps of alternation are shown in (**a**) the first entrance into the arm 1; (**b**) the second entry into the arm 3; and (**c**) the third entry into the arm 2 [41,42].

To assess the short-term memory (amnesic) effect, the exploratory activity of mice were investigated using Y- maze apparatus [39]. At a time, a single mouse was placed at Y-shaped maize and allowed to move freely for 5-min session and alterations were recorded as shown in Fig. 2. The percentage alternations were calculated as per reported protocol and mathematically as given in equation (2) [40].

[2] $$\%\;\text{Alternation}=\frac{S\text{pontaneous}\;\text{alternation}}{\text{Total}\;\text{number}\;\text{of}\;\text{arm}\;\text{entries}-\;2}\times 100$$

##### 2.4.3.2 Novel object recognition test (NORT)

**Fig. 3:**
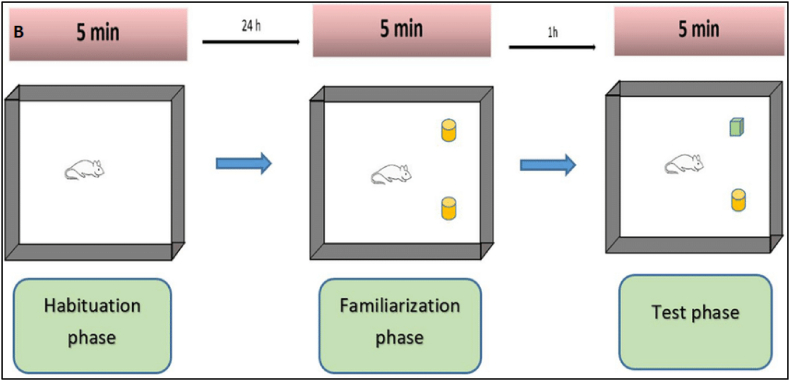
Procedures for novel object recognition (NORT). The three phases of the test are shown. In NORT, object identity is changed between training and test; object location is remained unchanged [45].

NORT was used to assess the effect of vanillin derivatives on memory in mice model. The apparatus employed in this test measures 40 cm wide, 40 cm long, and 66 cm tall. NORT entails a two-day habituation period, followed by a two-day training session and finally a testing or experimental period [43]. During habituation, an empty box was used, two identical objects were placed in two corners of the box during training, and one unique object was replaced during testing as shown in Fig. 3. To test long-term memory in mice, a five-day washout period was provided before the NORT. The time spent in each phase was manually documented using a stopwatch [44].

## 3 Results

### 3.1 *In-vitro* inhibition of AChE and BChE

The *in-vitro* concentration dependent inhibitions of AChE and BChE by using the synthesized compounds are displayed in Table 1. Almost all of the compounds were found active against both of the enzymes. The **SB-1** demonstrated the highest inhibitory activity against AChE (IC_50_ = 0.078 μM) whereas **SB-3** against BChE with IC_50_ of 0.057 μM respectively. Compound **SB-4** showed IC_50_ of 0.108 μM against AChE. Similarly, compound **SB-6** with IC_50_ of 0.045 μM value was also quite active against AChE. Compound **SB-5** and **SB-2** showed activity against AChE with IC_50_ of 0.442 μM and 0.610 μM respectively. Similarly, the BChE inhibition by compounds **SB-6**, **SB-5**, **SB-4**, **SB-3, SB-2** and **SB-1** in terms of IC_50_ values of 0.117 μM, 0.186 μM, 0.367 μM, 0.057 μM, 0.188 μM, and 0.265 μM was observed against BChE, respectively. The IC_50_ against cholinesterase by positive control galantamine at highest concentration was 0.014 μM and against BChE, with an IC_50_ value of 0.016 μM respectively.

**Table 1:**
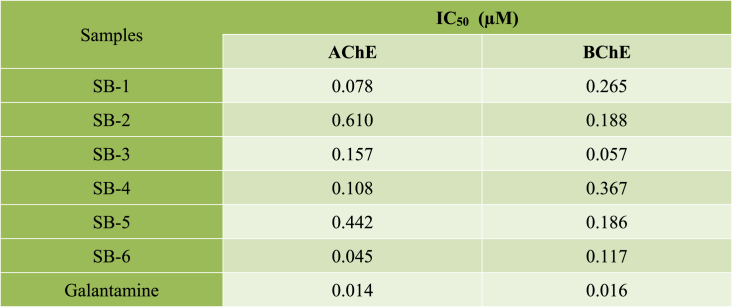
*In-vitro* AChE and BChE inhibitory assay. IC_50_ of SB1-SB6 in μM. The values are taken in triplicate. The data is presented in μM (n = 3).

### 3.2 Antioxidant potentials

The antioxidant potentials of synthesized compounds **SB1-SB6** have been determined against DPPH and ABTS. The results showed that almost all the compounds demonstrated a dose dependent response against the tested free radicals as shown in Table 2. In DPPH assay, the **SB-3** showed highest activity with IC_50_ of 0.305 μM followed by **SB-2** and **SB-1** with IC_50_ of 0.447 and 0.588 μM. The compounds **SB-4** and **SB-5** showed inhibition with IC_50_ value of 0.804 and 0.720 μM followed by **SB-6** having DPPH free radical inhibition with IC_50_ value of 2.803 μM. The compound's percent DPPH inhibition was compared with ascorbic acid (positive control) with IC_50_ of 0.074 μM.

**Table 2:**
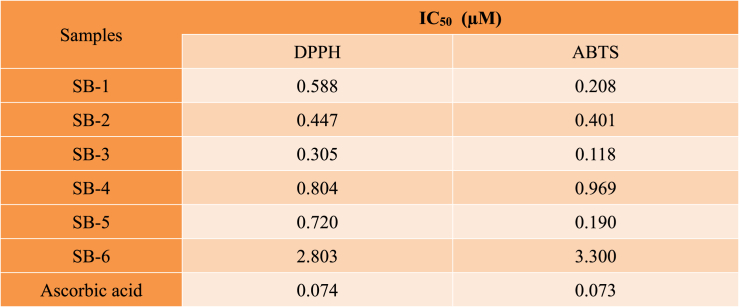
Percent DPPH and ABTS free radical scavenging activity of **SB1-SB6**. The values are taken in triplicate. The data is presented as μM (n = 3).

The percent inhibitions of **SB1-SB6** against ABTS free radical are also summarized in Table 2. Here maximum activity was recorded for compound **SB-3** followed by compound **SB-5** with IC_50_ values of 0.118 and 0.190 μM respectively. The compounds **SB-1**, **SB-2** and **SB-4** showed ABTS inhibition with IC_50_ value of 0.208, 0.401 and 0.969 μM respectively. Compound **SB-6** has poor activity against ABTS free radicals with IC_50_ value 3.300 μM was the least effective one. The percent ABTS inhibition by test compounds **SB1-SB6** were compared with ascorbic acid (positive control). Ascorbic acid showed inhibition against ABTS free radicals with IC_50_ of 0.073 μM.

### *3.3* *In vivo* studies

#### 3.3.1 Acute toxicity study results

**Fig. 4:**
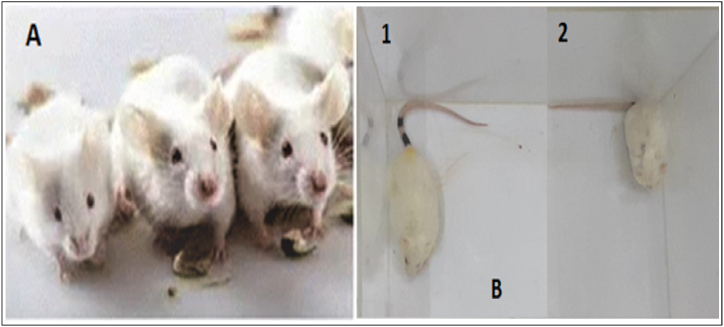
(**A**) Animals with normal behaviours receiving dose up to 1000 mg/kg body weight. (**B**) The acute toxic effect of the synthesized compounds on mice after 24 h at 2000 mg/kg dose.

Mice administered with synthetic compounds up to a dose of 1000 mg/kg, p.o. exhibited normal behaviour. They had typical grooming, touch, and pain responses and were awakened. Passivity, stereotypes, or vocalisation were not present. The secretory signs and motor activity were both normal as well. The animals didn't exhibit any depressive symptoms. The animal's alertness, limb tone, grip strength, and stride were all normal. It was discovered that the compounds were safe up to a dose of 1000 mg/kg body. Further increase in the dose by 50 percent altered the behaviour of animals after 20–24 h and induced salivation in some mice with decrease in water and food intake. Increase in urination, decrease in motor activities, alertness and sign of depression appeared in 40% of animals. While double fold increase in the last tested dose enhanced the toxic effects and lethality was observed in 35% of the test animals in the group receiving 1250 mg/kg dose. Over all study revealed that the compounds are safe up to the dose of 1000 mg/kg body weight as shown in Fig. 4. According to OECD guidelines, 100 mg/kg body weight and less were administered in the subsequent *in vivo* studies as it was 1/10th of the safe dose.

#### 3.3.2 Anti-alzheimer potential of test compound on Y maze test

**Fig. 5:**
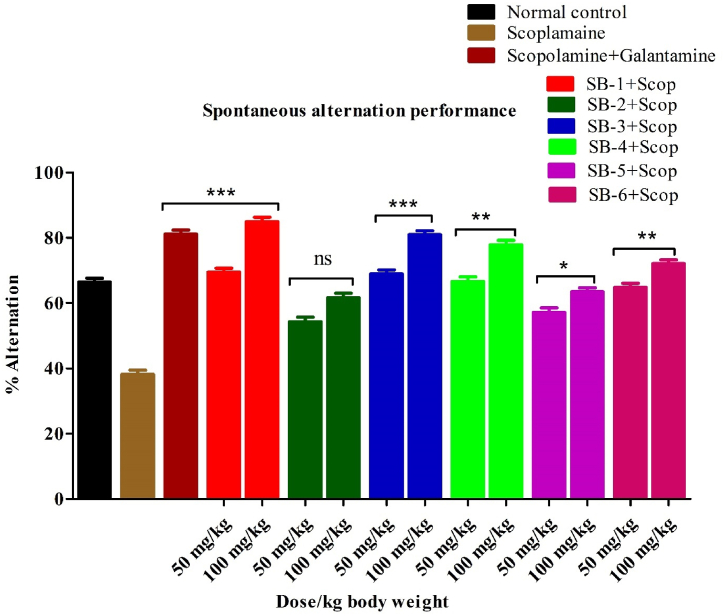
Effects of test compound **SB-1-SB6** on spontaneous alternation behaviour in Y-Maze. All data were analysed by one-way ANOVA and Dunnett's test and expressed in mean ± SEM (n = 6). Scopolamine-administered group was equated with saline treated group, while the standard and test groups were compared with the scopolamine-injected group. The significance values are marked as (P < 0.001) ***, (P < 0:01) ** and (P < 0.05) *.

**Fig. 6:**
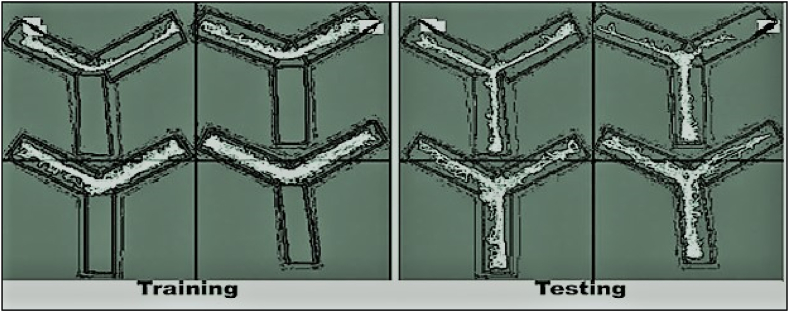
The figure shows the movement of the mice in the Y-maze, the frequency of entrance to the arms during the training and testing session.

Fig. 5 shows the result of the experiment that investigated the anti-amnesic effects of test compounds **SB-1** to **SB-6** in the Y-Maze test. The compounds were tested at a dose of 50 and 100 mg/kg body weight, along with the standard drug galantamine. The results showed that **SB-1** and **SB-3**, at both doses, significantly improved short-term memory retention in the Y-Maze test, as indicated by the 69.44 ± 1% (n = 6) and 84.88 ± 1% (n = 6) spontaneous alternation rates, respectively. This suggests that these compounds have potential anti-amnesic effects. In contrast, the scopolamine-treated group showed a significant impairment of memory, as indicated by the much lower 38.16% ± 1 (n = 6) spontaneous alteration rate compared to the normal control group. Furthermore, the standard group treated with galantamine and the test compounds **SB-1** and **SB-3** at a dose of 100 mg/kg b.w showed a significantly higher (P < 0.001) spontaneous alteration rate more than 80% compared to the scopolamine-treated group, indicating that they improved memory retention in the Y-Maze test. The movement of the mice in the Y-Maze and the frequency of entrance to the arms during the training and testing sessions are also shown in Fig. 6. The results suggest that **SB-4** and **SB-6** were more effective (P < 0.01) than **SB-5** (P < 0.05), while **SB-2** had no significant effect throughout the test period.

Overall, the results suggest that the test compounds **SB-1** and **SB-3**, have potential anti-amnesic effects and may be effective in improving short-term memory retention as observed in the Y-Maze test.

#### 3.3.3 Effects of SB-1 to SB-6 on scopolamine-induced memory impairment in novel object recognition test

##### 3.3.3.1 Latency time

**Fig. 7:**
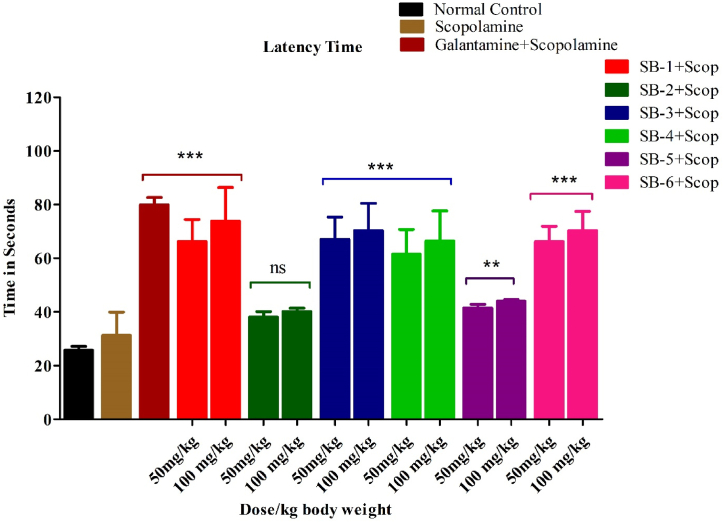
Effects of test compound on latency time during the novel NOR test. All data were analysed by one-way ANOVA and Dunnett's post hoc multiple comparison test and expressed in mean ± SEM (n = 6). Scopolamine-administered group was equated with saline treated group, while the standard and test groups were compared with the scopolamine-injected group. The significance values are marked as (P < 0.001) ***, (P < 0:01) ** and (P < 0.05) *.

In Fig. 7 the results of NOR test involving a Scopolamine group and a control group treated with galantamine. The study measured the latency time to discover a familiar object (F) and evaluated the effects of compounds (**SB-1, SB-2, SB-3, SB-4, SB-5, and SB-6**) on the latency time. There is a significant increase in the latency time for the Scopolamine group compared to the control group treated with galantamine (p < 0.001). This suggests that Scopolamine impaired the ability to recognize the familiar object F. Also, the administration of Compounds **SB-1, SB-3, SB-4, and SB-6** at both doses (50 and 100 mg/kg b.w) decreased the latency time significantly (p < 0.001). This indicates that these compounds improved the ability to recognize the familiar object F, possibly by counteracting the effects of Scopolamine. Compound **SB-5** showed a significant decrease in the latency time (p < 0.01), suggesting that it also had a positive effect on object recognition, although to a slightly lesser extent than the other compounds. In contrast, the compound **SB-2** remained insignificant, meaning that it did not have a significant effect on latency time.

##### 3.3.3.2 Exploration time of different objects

**Fig. 8:**
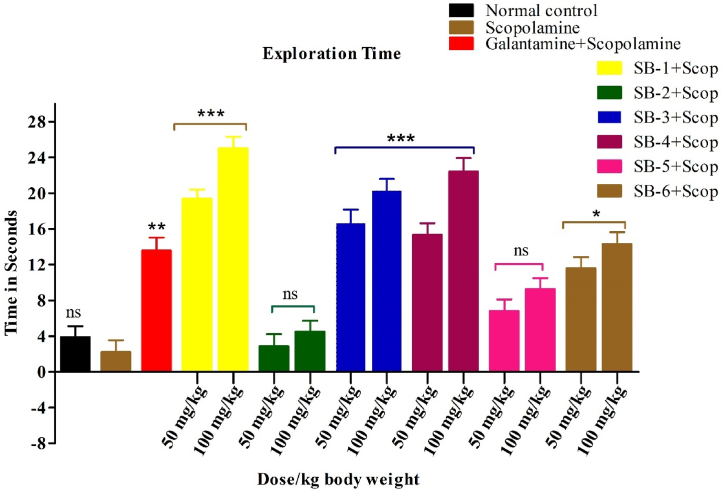
Effects of test compound on exploration time in the novel NOR test. All data were analysed by one-way ANOVA and Dunnett's post hoc multiple comparison test and expressed in mean ± SEM (n = 6). Scopolamine-administered group was equated with saline treated group, while the standard and test groups were compared with the scopolamine-injected group. The significance values are marked as (P < 0.001) ***, (P < 0:01) ** and (P < 0.05) *.

The test estimates the time spent by mice with a familiar object (F) and a new object (N) after being treated with Scopolamine, Galantamine and test compounds **(SB-1, SB-2, SB-3, SB-4, SB-5, and SB-6)**. Fig. 8, presents the results of the study, indicating a significant increase (P < 0.001) in the time spent with the familiar object F for the Scopolamine group compared to the control group treated with galantamine, being standard drug.

On the hand, administration of compounds **SB-1, SB-3, and SB-4** at the administered doses of 50 and 100 mg/kg b.w resulted in a significant increase (P < 0.001) in the time spent with the new object N, indicating that these compounds improved the ability of mice to recognize and explore a new object. In contrast, **SB-2 and SB-5** showed insignificant activity at both doses compared to the Scopolamine group, suggesting that they did not improve object recognition. The group receiving galantamine also showed a significant increase (P < 0.001) in the time spent with the new object N, indicating its effectiveness in improving object recognition in mice. The compound **SB-6** showed slight activity (P < 0.05), suggesting that it had a limited effect on object recognition compared to the other tested compounds.

Overall, the results suggest that the tested compounds have varying degrees of effectiveness in improving object recognition in mice. However, **SB-1, SB-3, and SB-4** showed the most significant activity, while **SB-2 and SB-5** showed insignificant activities.

##### 3.3.3.3 Discrimination index (%)

**Fig. 9:**
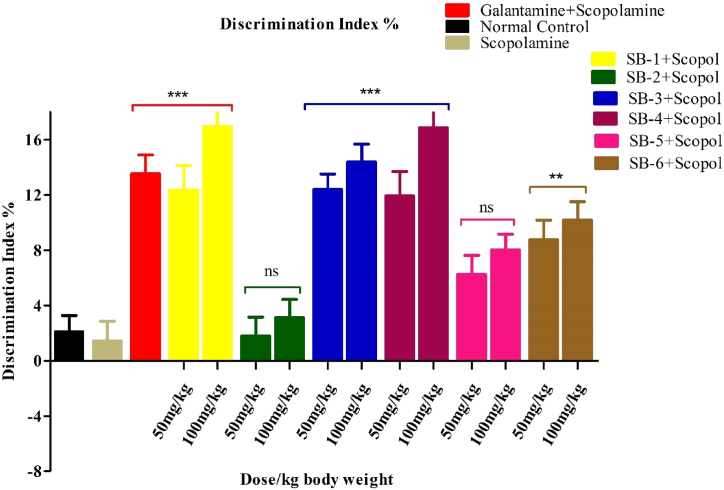
Effects of test compound on Discrimination index percentage in the NOR test. All data were analysed by one-way ANOVA and Dunnett's post hoc multiple comparison test and expressed in mean ± SEM (n = 6). Scopolamine-administered group was equated with saline treated group, while the standard and test groups were matched with the scopolamine-injected group. The significance values are marked as (P < 0.001) ***, (P < 0:01) ** and (P < 0.05) *.

The discrimination index in mice after the administration of the compounds **(SB-1 to SB-6**) treated with Scopolamine and Galantamine was also evaluated. The discrimination index measures the ability of mice to distinguish between a familiar object and a new object. Fig. 9, presents the discrimination index of the familiar object for mice in the Scopolamine-treated group (1.43% ± 1) compared to the control group treated with Galantamine (13.54% ± 1) indicating that the impairment in the case of scopolamine is higher than in the standard drug treated group.

All the administered compounds at both doses; 50 mg/kg and 100 mg/kg b.w have resulted in a significant increase (p < 0.001) in the discrimination index, except for **SB-2 and SB-5**, which had exhibited insignificant activity. This indicates that these compounds can improve the ability of mice to discriminate between familiar and new objects by counteracting the effects of Scopolamine. The **SB-1, SB-3, and SB-4** showed the most prominent activity at both doses when compared to Galantamine group. The activity of **SB-1 and SB-4** was equivalent, as both compounds showed nearly the same range of activity. This suggests that these compounds have the ability to increase the memory level of amnesic mice, possibly even more effectively than Galantamine. Overall, the results suggest that the tested compounds have potential as therapeutic agents for improving memory and cognitive function, particularly in conditions of cognitive impairment or amnesia.

## 4 Discussion

Memory is the process where experiences are documented in brain that can be used to acclimate their responses to the environment [46]. It has great importance in some one life to define itself and their place along with normal working life. Central cholinergic system is considered to be the most important neurotransmitter involved in regulation of cognitive functions [47]. Acetyl cholinesterase is the key enzyme responsible for acetylcholine hydrolysis which terminates the cholinergic transmission [48]. Decrease in the cholinergic conduction is associated with cognitive dysfunction and as reported in neurodegenerative diseases such as Alzheimer's disease [49]. Literature studies have revealed that cholinesterase inhibitors might act on several therapeutic targets such as AChE enzyme that is responsible for acetylcholine hydrolysis [50]. Though many inhibitors of AChE are available in market but none of them are 100% efficient. Therefore, scientific search is continued to discover efficient novel AChE inhibitors with low toxicity and high penetration rate to the central nervous system. The standard drugs used as acetyl cholinesterase inhibitors in treating AD are galantamine, donepezil and rivastigmine. AChE inhibition is also considered as a remedial strategy for other types of neuronal disorders like dementia and Parkinson's diseases. Many Schiff bases synthesized in various parts of the world have been evaluated for anti-amnesic and anti-Alzheimer's activity with excellent activities [51].

In the present study, the vanillin analogues prepared was found potent to inhibit the Acetyl cholinesterase and butyryl cholinesterase enzymes and thus were able in restoring the memory deficit caused by scopolamine. The inhibition of cholinesterases tends to allow more retention of acetylcholine in the brain, which is important for the cognitive function, learning and memory.

Oral suspension of the compounds was fed to the mice models to evaluate memory and learning enhancing effects of the compounds via Y maze and NORT. The *in-vivo* behavioural study confirmed the association of cholinesterases (AChE and BChE) activities with memory enhancement in mice. Y-maze test is based on the willing of the animals to explore new environment. The normal animals will explore the new arm; the animals whose memory is not working properly will again enter the old arm previously explored. The infected animals gave a smaller number of spontaneous explorations as compared to normal animals. In this study, all compound except **SB-2** and **SB-5** significantly decreased the number of alternate arms returns as compared to control. Furthermore, the compounds also significantly increased spontaneous alternation performance which was parallel with standard drug.

Novel object recognition test was used for studying both short- and long-term memory. The general principle of novel object recognition test is based on exploring new object. The rodents spend more time with unfamiliar object as compared to familiar object. Compounds **SB-1** to **SB-6** caused increase exploration of the novel object compared to the familiar object whose results were comparable to reference drug galantamine. The compound **SB-2** did not cause any changes in the memory and cognition of mice.

In NORT, the increase of the %DI and novel object exploration time in mice treated with vanillin analogues suggested an improvement of learning and memory in mice that was reduced by scopolamine. The effect of scopolamine was also reversed by all compound significantly as increase in %DI and novel object exploration time were recorded, indicating that vanillin analogues possessed memory enhancing activity. These findings were further supported by their potential to inhibit AChE and BChE enzymes both *in-vitro*.

AChE and BChE are enzymes that break down the neurotransmitter acetylcholine in the brain, which plays an important role in learning and memory. Inhibiting these enzymes leads to increased levels of acetylcholine in the brain, which can improve cognitive function. It was found that the vanillin analogues are able to inhibit both AChE and BChE enzymes potently. The results obtained were in close agreement with the reported study [47]. The results of *in-vivo* studies suggest that the analogues could cross the blood-brain barrier and inhibit the activity of these enzymes in the brains of living organisms.

The results suggest that the memory-enhancing effects of the vanillin analogues may be in part or totally due to their ability to inhibit AChE and BChE enzymes. Inhibition of these enzymes could lead to increased acetylcholine levels in the brain, which may help in improving memory and cognitive function.

## 5 Conclusions

Herein, vanillin analogues were synthesized to explore their antioxidant and anti-amnesic potentials both *in-vitro* and *in-vivo*. The antiamnesic potential was evaluated through marker enzymes: AChE and BChE whereas antioxidant potential was evaluated against DPPH and ABTS free radicals. The compounds; **SB-1**, **SB-3**, **SB-4**, and **SB-6** were found to be good inhibitors of AChE and BChE respectively whereas almost all the compounds were potent antioxidants. In *in-vivo* studies in Y-Maze and new object recognition test exhibited a considerable decline in cognitive dysfunctions. These compounds could be used as antiamnesic agents and drug candidate of treating oxidative stress however, further experiments in other animal models are required to fully confirm the observed biological effects in this study.

## Data availability

No data was used for the research described in the article.

## CRediT authorship contribution statement

**Qamar Gul:** Writing – original draft, Methodology, Conceptualization. **Nasiara Karim:** Writing – original draft, Methodology, Conceptualization. **Mohammad Shoaib:** Writing – original draft, Methodology, Conceptualization. **Muhammad Zahoor:** Writing – review & editing, Writing – original draft, Methodology, Conceptualization. **Mehboob Ur Rahman:** Writing – original draft, Methodology, Formal analysis. **Hayat Bilal:** Writing – original draft, Validation, Formal analysis, Data curation. **Riaz Ullah:** Writing – original draft, Resources, Methodology, Formal analysis, Data curation. **Amal Alotaibi:** Writing – original draft, Resources, Methodology, Investigation, Formal analysis, Data curation.

## Declaration of competing interest

The authors declare that they have no known competing financial interests or personal relationships that could have appeared to influence the work reported in this paper.
